# Prevalence of proliferating CD8^+^ cells in normal lymphatic tissues, inflammation and cancer

**DOI:** 10.18632/aging.203113

**Published:** 2021-06-03

**Authors:** Niclas C. Blessin, Raed Abu-Hashem, Tim Mandelkow, Wenchao Li, Ronald Simon, Claudia Hube-Magg, Christina Möller-Koop, Melanie Witt, Alice Schmidt, Franziska Büscheck, Christoph Fraune, Andreas M. Luebke, Katharina Möller, Frank Jacobsen, Florian Lutz, Maximilian Lennartz, Stefan Steurer, Guido Sauter, Doris Höflmayer, Maria Christina Tsourlakis, Andrea Hinsch, Eike Burandt, Waldemar Wilczak, Sarah Minner, Till S. Clauditz

**Affiliations:** 1 Institute of Pathology, University Medical Centre Hamburg-Eppendorf, Hamburg D-20246, Germany

**Keywords:** tumor infiltrating lymphocytes, CD8+ cytotoxic T cells, tumor microenvironment, lymphatic tissue, colorectal cancer

## Abstract

CD8^+^ cytotoxic T-lymphocytes are essential components of the anti-tumor immunity. To better understand the expansion of CD8^+^ T-cells we used multiplex fluorescence immunohistochemistry to study Ki67^+^CD8^+^ cells in normal lymphoid tissues, selected inflammatory diseases and cancers in 41 large sections/ microenvironment tissue microarrays (TMAs) as well as 765 samples in a conventional TMA format. The evaluation of more than 20 different compartments of normal lymphoid tissues revealed that the percentage of proliferating (ki67^+^) CD8^+^ cells did commonly not exceed 3%. In inflammations, the percentage of Ki67^+^CD8^+^ cells was more variable and higher compared to normal tissues. In cancers, the percentage of Ki67^+^CD8^+^ cells was higher in the tumor center than at the invasive margin. In the tumor center of 765 colorectal cancers, the density of Ki67^+^CD8^+^ cells and the percentage of proliferating CD8^+^ cytotoxic T-cells was significantly associated with microsatellite instability (p<0.0001), pT (p<0.0002) and pN category (p<0.0098). In summary, these data show that the percentage of Ki67^+^CD8^+^ cells is usually at a baseline proliferation rate below 3% in healthy secondary lymphoid organs. This rate is often markedly higher in inflammatory and neoplastic diseases compared to normal tissues. The striking link with unfavorable tumor features in colorectal cancer suggest a potential clinical utility of assessing the percentage of Ki67^+^CD8^+^ cells to predict patients outcome.

## INTRODUCTION

Tumor infiltrating lymphocytes (TILs) are of topical interest. TILs occur at variable frequency in cancers [1]. For many tumor types, a high number of TILs has been shown to be linked to a favorable prognosis [2–7]. The striking success of immune checkpoint inhibitors targeting PD-1, PD-L1, CTLA-4 and other modulators of the immune reaction in various tumor entities such as melanoma, non-small-cell lung cancer, kidney cancer and bladder cancer have raised the interest in the composition and activity of the tumor infiltrating lymphocytes such as the proliferating subset of cytotoxic T-cells [8, 9]. It is generally assumed that CD8^+^ cytotoxic T-lymphocytes represent the most important TIL subset which can directly kill cancer cells [10–12]. High CD8^+^ T-cell densities are found to be associated with favorable prognosis in a rapidly raising number of tumor entities, for example melanoma, colorectal cancer, breast cancer, hepatocellular cancer and non-small cell lung cancer [2, 5, 11–14]. However, presence of CD8^+^ T-cells in the tumor microenvironment does not necessarily mean that these cells are actively killing cancer cells, because most cytotoxic T-cells are non-tumor antigen specific bystander T-cells [15, 16]. The degree of CD8^+^ T-cell activation is modulated by an, as to yet, not fully understood immune regulatory network including immune checkpoint molecules. It is, thus, desirable to not only quantitate CD8^+^ T-cells in the cancer microenvironment but to also measure distinct CD8^+^ T-cell subsets, such as active and expanding CD8^+^ T-cells [13, 17, 18]. The Ki67 protein can be utilized for this purpose. Ki67 is expressed in all proliferating cells in G1, S, G2, and M phase of the cell cycle [19, 20]. Thus, the Ki67^+^ expanding CD8^+^ T-cell subset has been shown to represent an activated and anti-tumor specific subset of CD8^+^ T-cells [15, 16]. While several studies have investigated Ki67^+^CD8^+^ cells in neoplasms and inflammatory diseases [17, 18, 21, 22], little is known about the prevalence and distribution of Ki67^+^CD8^+^ T-cells in normal lymphatic organs. To learn more on the sites of CD8^+^ T-cell proliferation under normal and pathological conditions, we analyzed a collection of tissues from normal lymphatic organs, selected inflammatory conditions and cancers in a tissue microarray format (TMA) as well as in conventional large sections using multiplex-fluorescence immunohistochemistry.

## RESULTS

### Ki67^+^CD8^+^ T-cells in healthy lymphatic tissue

**Figure 1:**
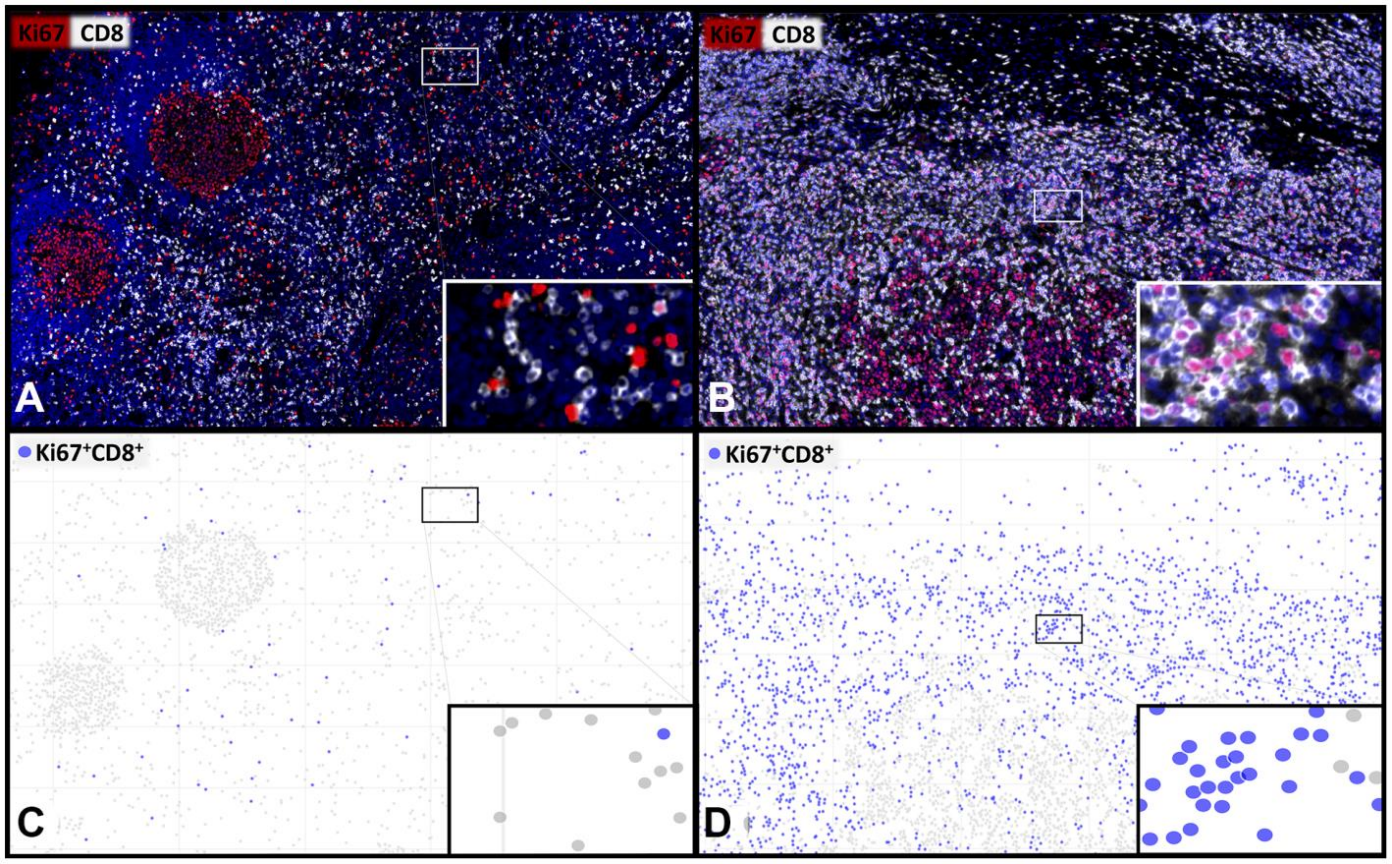
**Representative images and visualisations showing the cell detection of proliferating CD8^+^ T-lymphocytes.** Multiplex immunofluorescence images (**A**, **B**) showing CD8^+^ cytotoxic T-lymphocytes (white) and Ki67^+^ proliferating cells (red) with a low (**A**, **C**) and a high (**B**, **D**) percentage of proliferating Ki67^+^CD8^+^ T-cells. The visualization (**C**, **D**) of the digital image analysis underlines the accuracy of the automated detection of the subset of Ki67^+^CD8^+^ proliferating cytotoxic T-cells (blue). 400x magnifications are shown in the insets.

**Figure 2:**
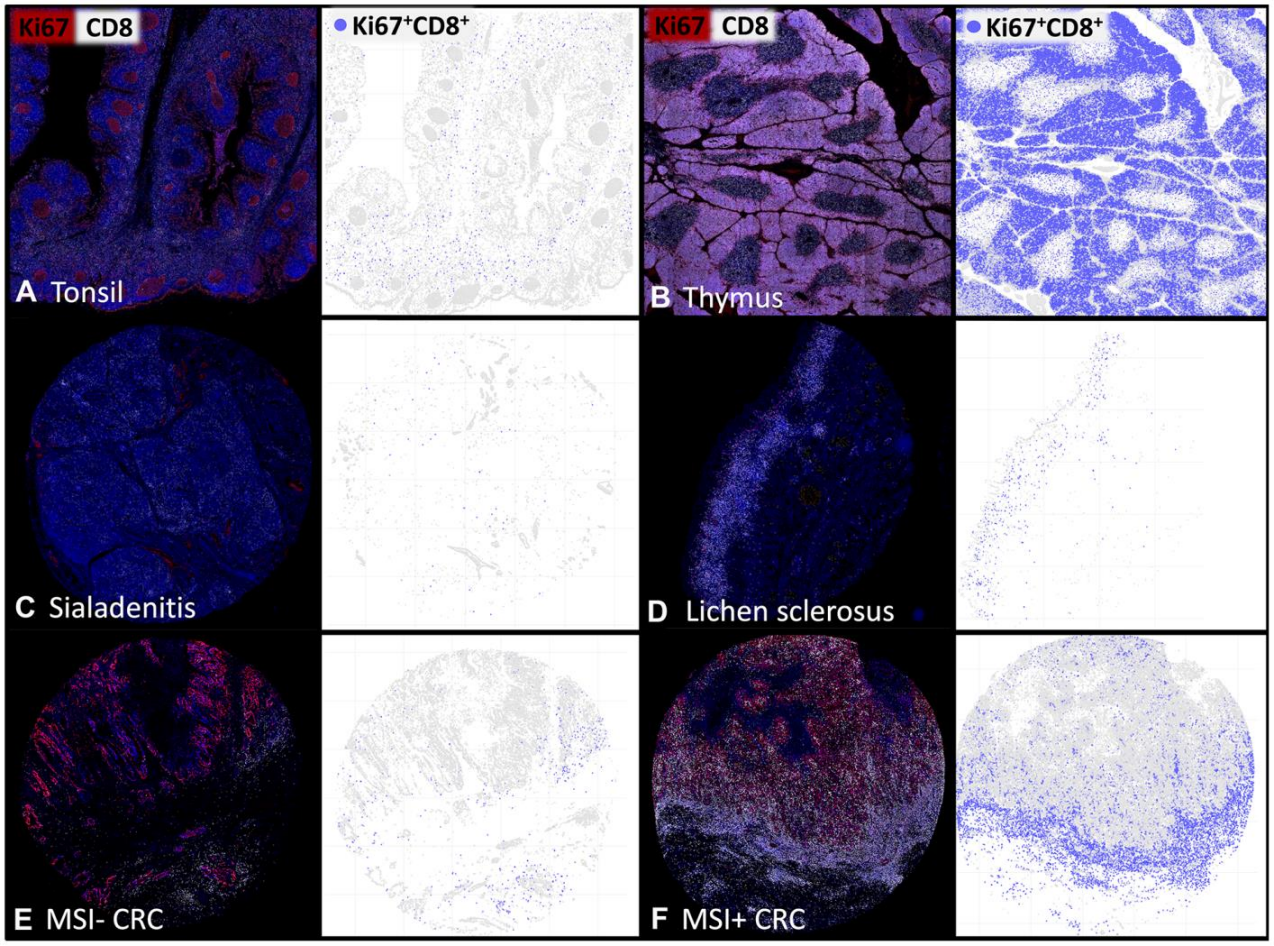
**The density of proliferating CD8^+^ T-lymphocytes varies between tissues and individual patients.** Representative multiplex immunofluorescence images of CD8^+^ (white) and Ki67^+^ (red) cells in (**A**) normal human tonsil, (**B**) thymus, (**C**) sialadenitis, (**D**) lichen sclerosus, (**E**) Microsatellite stable colorectal cancer (MSI- CRC) and (**F**) Microsatellite instable colorectal cancer (MSI+ CRC). The visualizations of the digital image analysis highlight the subset of Ki67^+^CD8^+^ proliferating cytotoxic T-cells (blue).

The analysis of normal lymphatic tissues revealed that the percentage of proliferating (ki67^+^) CD8^+^cells (Figure 1) varied slightly between organs and tissue compartments but typically did not exceed 3% (range 0% to 3.1%). For example, the percentage of proliferating CD8^+^ cells in normal lymph node and tonsil was 0-0.3% in the germinal centers, 0.8-2.3% in the marginal zone and 1.3-3.0% in the interfollicular zone (Figure 2A). Whereas the cortex of the thymus showed an exceptionally high percentage of proliferating CD8^+^ cells (45.2%, Figure 2B) and was thus excluded from correlation analysis and calculations of the overall mean. All data for normal lymphatic tissues are summarized in Table 1. The percentage of proliferating CD8^+^ cells was unrelated to the density of CD8^+^ cells in all analyzed normal tissues and their compartments (r=0.16; p=0.41, Supplementary Figure 2A) with the exception of thymus. It is of note, that the distribution and percentage of Ki67^+^CD8^+^ cells in lymph node metastasis did not show any differences compared to normal lymph nodes.

**Table 1:** Percentage of proliferating Ki67^+^CD8^+^ cells, the density of Ki67^+^CD8^+^ cells/mm^2^ and the density of CD8^+^ cells/mm^2^ in distinct histological compartments of healthy lymphatic tissue.

**Tissue**	**Compartment**	**Percentage of proliferating CD8^+^ cells**	**Density of CD8^+^Ki67^+^ cells [cells/mm^2^]**	**Density of CD8^+^ cells [cells/mm^2^]**
Thymus	Cortex	45.2	7350	16273
	Medulla	4.7	195	4119
Tonsil	Interfollicular area	1.3	30	2256
	- CD8^+^ rich	2.7	72	2693
	- perifollicular	1.6	21	1341
	- CD8^+^ intermediate	1.4	30	2187
	- superficial	1.3	6	457
	- CD8^+^ poor	1.0	7	770
	Marginal zone	0.8	3	310
	Germinal center	0.3	0	138
Lymphnode	Interfollicular area	3.0	65	2201
	Marginal sinus	2.3	30	1297
	Marginal zone	2.3	2	71
	Medulla	2.0	65	3257
	Germinal center	0.0	0	23
Spleen	Germinal center	3.1	8	267
	Marginal zone	2.1	3	157
	Red pulp	1.6	43	2798
	Perifollicular area	1.1	4	412
	Mantle zone	0.0	0	101
Peyer's patch (Ileum)	Mucosa	2.1	35	1641
	Interfollicular area	1.6	43	2699
	Submucosa	1.4	1	1388
	Marginal zone	1.1	28	2626
	Germinal center	0.0	0	64
Appendix	Mucosa	2.9	9	330
	Marginal zone	1.1	25	2255
	Germinal center	0.0	0	440
**All (±SD)**	**Thymus excluded**	**1.4 (±1.1)**	**21 (±29)**	**513 (±443)**

SD: Standard deviation.

### Ki67^+^CD8^+^ cells in inflammation

In inflammation, both the mean percentage of proliferating CD8^+^ cells and the mean density of CD8^+^ cells were higher and more variable compared to normal tissues (Figure 2C, 2D). For example, the percentage of Ki67^+^CD8^+^ cells ranged from 0.5% in one patient with sialadenitis to 18.8% in the intraepithelial compartment of Crohn’s disease (Table 2). The density of CD8^+^ cells ranged between 35 cells/mm^2^ in sialadenitis to 2365 cells/mm^2^ in Hashimoto thyroiditis (Table 2). Again, the proliferation rate was unrelated to the density of CD8^+^ T-cells in inflammations (r=-0.28; p=0.17; Supplementary Figure 2B).

**Table 2:** Percentage of proliferating Ki67^+^CD8^+^ cells, the density of Ki67^+^CD8^+^ cells/mm^2^ and the density of CD8^+^ cells/mm^2^ in distinct histological compartments of inflammations.

**Tissue**	**Compartment**	**Patient**	**Percentage of proliferating CD8^+^ cells**	**Density of CD8^+^Ki67^+^ cells [cells/mm^2^]**	**Density of CD8^+^ cells [cells/mm^2^]**
Crohn's	Intraepithelial	1	18.8	30	161
disease		2	17.8	34	192
	Subepithelial	1	1.3	22	1602
		2	4.7	43	924
Eczema	Intraepithelial	1	11.0	13	117
	Subepithelial	1	4.9	37	751
Hashimoto	Interfollicular area	1	3.8	90	2365
thyroiditis		2	10.3	104	1010
	Marginal zone	1	2.5	6	256
		2	5.0	27	534
	Germinal center	1	11.1	13	120
		2	12.5	28	224
Lichen	Intraepithelial	1	5.3	35	666
sclerosus		2	13.3	36	274
	Subepithelial	1	3.0	82	2690
		2	5.9	164	2777
Sarcoidosis	Granuloma	1	5.1	75	1468
		2	4.5	17	386
	Intergranuloma	1	7.1	14	204
		2	1.6	1	72
Pancreatitis	CD8^+^ rich	1	5.3	82	1563
		2	2.0	28	1362
	CD8^+^ poor	1	2.3	11	457
		2	2.6	2	62
Sialadenitis		1	1.0	3	252
		2	0.5	0	35
**All (±SD)**	****	****	**6.3 (±5)**	**38 (±38)**	**789 (±815)**

SD: Standard deviation.

### Ki67^+^CD8^+^ cells in cancer samples

The analysis of our cancer microenvironment TMA containing extra-large tissue samples suggested a high variability of both the percentage of Ki67^+^CD8^+^ cells and the density of CD8^+^ cells both between cancer types and between individuals (Figure 2E, 2F). Of note, the percentage of Ki67^+^CD8^+^ cells was mostly higher than the 3% found as the maximal value in normal lymphatic organs. The percentage of Ki67^+^CD8^+^ was the highest (Mean 20.6%) in colorectal carcinomas showing microsatellite instability (Table 3).

**Table 3:** Percentage of proliferating Ki67^+^CD8^+^ cells, the density of Ki67^+^CD8^+^ cells/mm^2^ and the density of CD8^+^ cells/mm^2^ in the center of the tumor and the invasive margin of various cancer entities.

**Cancer**	**Compartment**	**Patient**	**Percentage of proliferating CD8^+^ cells**	**Density of CD8^+^Ki67^+^ cells [cells/mm^2^]**	**Density of CD8^+^ cells [cells/mm^2^]**
Colon MSI positive	Invasive margin	1	20.9	309	1477
		2	21.8	319	1465
		3	16.2	782	4815
		4	24.0	268	1119
	Center of the tumor	1	22.3	389	1747
		2	20.0	403	2013
		3	17.3	423	2447
		4	30.7	651	2119
		5	12.3	10	80
Colon MSI negative	Invasive margin	1	3.3	24	714
		2	6.1	57	934
		3	3.3	30	886
		4	2.3	1	40
		5	10.3	83	809
	Center of the tumor	1	17.6	10	55
		2	14.3	16	111
		3	5.3	11	208
		4	8.2	1	7
		5	23.7	18	75
Bladder	Invasive margin	1	0.5	5	973
		2	3.6	1	39
	Center of the tumor	1	1.5	5	308
		2	15.3	5	31
Lung	Invasive margin	1	0.9	1	82
	Center of the tumor	1	0.0	0	20
		2	12.8	27	211
Lymphnode metastases	Interfollicular area	1	2.1	33	1607
		2	2.3	78	3300
	Invasive margin	1	3.2	37	1142
		2	3.0	64	2313
	Center of the tumor	1	9.0	4	50
		2	3.0	2	56
Melanoma	Invasive margin	1	0.0	0	47
	Center of the tumor	1	0.0	0	3
		2	11.1	0	1
Prostate	Invasive margin	1	0.0	0	12
	Center of the tumor	1	0.0	0	11
		2	4.7	1	14
Kidney	Invasive margin	1	0.0	0	12
	Center of the tumor	1	0.6	0	30
		2	3.4	0	7
**All (±SD)**	****	****	**8.7 (±8.5)**	**99 (±186)**	**766 (±1064)**

SD: Standard deviation.

### Ki67^+^CD8^+^ cells in colorectal cancer

The CD8^+^/Ki67^+^ measurement was successful in 765 (93%) of the 826 0.6mm tissue spots of the colorectal cancer TMA. The remaining 61 (7%) spots were excluded due to a lack of tissue or absence of unequivocal cancer cells. The density of CD8^+^ T-cells ranged from 0 to 4,273 cells/mm^2^ (mean:330 cells/mm^2^) and the density of Ki67^+^CD8^+^ cells ranged from 0 to 3,238 cells/mm^2^ (mean: 89 cells/mm^2^), while the percentage of proliferating CD8^+^ T-cells ranged from 0 to 100% (mean: 20.6%). All unfavorable clinicopathological parameters (e.g., increased tumor stage, positive nodal stage and microsatellite status) were significantly associated with a low density and a low percentage of proliferating CD8^+^ T-cells (p≤0.01 each; Table 4).

**Table 4:** Association between the percentage of proliferating Ki67^+^CD8^+^ cells, the density of Ki67^+^CD8^+^ cells/mm^2^ as well as the density of CD8^+^ cells/mm^2^ and the histopathological phenotype of 765 colorectal cancer samples.

**Clinical parameter**	**n**	**Percentage of proliferating CD8^+^ cells (±SD)**	**p-value**	**Density of CD8^+^Ki67^+^ cells [cells/mm^2^] (±SD)**	**p-value**	**Density of CD8^+^ cells [cells/mm^2^] (±SD)**	**p-value**
pT1	36	31.5 (±21.6)	<0.0001	218 (±304)	0.0002	808 (±1258)	<0.0001
pT2	151	27.4 (±24.1)		120 (±315)		384 (±631)	
pT3	411	19.8 (±21.2)		83 (±231)		294 (±294)	
pT4	167	15.6 (±19.1)		45 (±107)		264 (±413)	
pN-	390	23.6 (±23.0)	0.0005	108 (±274)	0.0098	383 (±653)	0.0064
pN+	372	18.1 (±20.1)		64 (±181)		267 (±504)	
MSI-	525	23.3 (±21.7)	<0.0001	91 (±248)	<0.0001	308 (±528)	0.0009
MSI+	32	42.5 (±26.1)		314 (±450)		638 (±777)	

pT: pathological tumor stage; pN: pathological nodal stage; MSI: microsatellite instability.

## DISCUSSION

The separate analysis of more than 20 different compartments of healthy secondary lymphoid organs enabled us to identify a general upper limit of the percentage of proliferating (ki67^+^) CD8^+^ cytotoxic T-lymphocytes ranging between 1% and 3% in normal tissues. This is consistent with low proliferation rates described in previous studies using different technologies [23–25]. For example, Golby et al. used multiplex brightfield immunohistochemistry to study the percentage of proliferating CD8^+^ T-cells in the interfollicular compartment of 5 tonsils as well as 6 Peyer’s patches and described CD8^+^ proliferation rates below 1% [24]. Lempicki et al. revealed also a mean percentage of proliferating CD8^+^ T-cells below 1% in blood samples of 67 healthy patients measuring the percentage of proliferating cells with BrdU uptake by flow cytometry [25]. The strikingly high rate of Ki67^+^CD8^+^ cells in the thymus fits well with its unique role in T-cell expansion [26–28].

The spectrum of analyzed inflammatory diseases and the number of patients included in this study was too limited for drawing major conclusions on the specific role of Ki67^+^CD8^+^ cells in inflammation. It was conspicuous, however, that inflamed tissues showed a higher and much more variable percentage of proliferating CD8^+^ cells than normal tissues. The increase of Ki67^+^CD8^+^ cells in inflammatory conditions fits well to the concept that expansion of cytotoxic T-cells is driven by a proinflammatory cytokines-rich microenvironment [16, 29–31]. Among 7 analyzed inflammatory diseases, the percentage of CD8^+^ cytotoxic T-cell proliferation ranged from 0.5% to 18.8% and the density of Ki67^+^CD8^+^ cells ranged from 0 cells/mm^2^ to 164 cells/mm^2^. The high variability of parameters for CD8^+^ cytotoxic T-cell proliferation suggests that quantitating cytotoxic T-cell expansion can serve as a useful parameter for the characterization of inflammatory diseases: A recent study reported that a significantly elevated CD8^+^ cytotoxic T-cell infiltration in ileal lamina propria was accompanied by early endoscopic recurrence in Crohn’s disease [32] and another chronic inflammatory bowel disease – microscopic colitis – showed an increased proportion of proliferating CD8^+^ T-cells compared to controls [33]. In addition, elevated CD8^+^ T-cell proliferation was recently detected in the context of dermatologic immune-related adverse effects caused by anti-PD-1 therapy [34].

In a total of 5 cases with skin eczema, Crohn’s disease and lichen sclerosus, the intraepithelial and subepithelial compartment could be separately evaluated. The fact that the percentage of Ki67^+^CD8^+^ cells was always higher within the epithelial cell layer than in the adjacent subepithelial stroma could suggest that the epithelium represents the epicenter of the immunologic reaction in these diseases. This is a particular good example for localization specific differences in T-cell accumulation and proliferation in an inflamed immune environment, which is well known in another inflammatory disease: The Hashimoto thyroiditis is characterized by abundant lymphocytic infiltration including large numbers of B-lymphocytes forming particularly large but normal appearing secondary lymph follicles. In an earlier study evaluating the expression of PD-1 and T-cell immunoglobulin and ITIM domain (TIGIT) in normal and diseased tissues we had found the highest TIGIT and PD-1 levels among CD4^+^ and CD8^+^ T-lymphocyte located in the germinal centers of Hashimoto thyroiditis [35]. The strikingly higher percentage of Ki67^+^CD8^+^ cells in Hashimoto lymph follicles than in normal tonsil or lymph follicles further demonstrates the peculiar role of these lymph follicles.

To study the role of Ki67^+^CD8^+^ cells in cancer, two separate types of tissue microarrays were used in this project. A total of 20 cancers were analyzed in a “microenvironment TMA” containing extra-large TMA spots with a diameter of 4 mm. This sample size is usually sufficient to evaluate epithelial-stroma interactions. These samples were taken from the tumor periphery to enable a separate analysis of the invasive margin and of more centrally located tumor areas. That the percentage of Ki67^+^CD8^+^ cells was typically higher in the center than at the invasive margin in 12 cancers where both tissue compartments could be assessed is consistent with CD8 expansion occurring in the immediate proximity of the projected field of action. Given that the proliferative activity of CD8^+^ cells in the analyzed healthy tissues was below 3% in 20 of 21 interpretable compartments, one might even speculate that proliferative stimuli are often minimal in this area compared to some inflamed cancer microenvironments.

The second cancer TMA contained one 0.6mm sample each from 765 different colorectal cancers. These samples were not selectively taken from the tumor periphery and thus only enabled to quantitate lymphocyte subpopulations in the tumor center. This TMA was used to compare numbers of Ki67^+^CD8^+^ cells with parameters of cancer aggressiveness in one distinct cancer entity. The significantly higher rates of CD8^+^ cells in cancers with microsatellite instability and the association between high CD8 cell count and favorable tumor features such as low stage and absence of nodal metastases fits well with the existing literature [18, 36]. These findings also validate our conventional TMA approach utilizing one tissue core of 0.6mm in diameter for assessing at least certain aspects of the microenvironment of colorectal cancers.

The striking association of abundant Ki67^+^CD8^+^ cells with favorable tumor features such as low pathological tumor stage and absence of nodal metastases is consistent with a pivotal biological and clinical role of the magnitude of CD8 expansion in colorectal cancer. It is not counter-intuitive that the immune system’s capacity to continuously replace exhausted cytotoxic T-cells must be crucial for cancer control. One might even hypothesize that immunologic tumor control could depend on the ratio between tumor cell expansion and immunologic tumor killing. Tumor cell expansion of colorectal cancer cells has been extensively studied and it was shown to be highly variable: The Ki67 labeling index (LI) of colorectal cancer ranged between 10% and 85% (mean 30% to 44%) in various studies [37–40]. Analyzing the dynamics of cytotoxic T-cell mediated killing of virus infected cells by two-photon imaging revealed that the killing capacity of CD8^+^ T-cells is limited to 2-16 diseased cells per day [10]. The strong correlation between the proliferative activity of CD8^+^ T-cells and the absolute number of CD8^+^ T-cells seen in a tumor demonstrates that local expansion is a major determinant for cytotoxic T-cell abundancy in a tumor. In addition, the fact that exceedingly high local CD8^+^ T-cell proliferation was found in compartments of both colorectal cancer and its corresponding inflammatory disease also underlines the important role of local T-cell expansion in the cancer microenvironment, as well known from chronic inflammatory bowel diseases [41, 42].

We consider it as a strength of this study that all our findings were precisely quantitated using reproducible numerical parameters such as the percentage of Ki67^+^CD8^+^ cells as well as the density of Ki67^+^CD8^+^ and CD8^+^ cells (per mm^2^). The use of measurable numerical parameters facilitates comparison of data from different studies. The density of Ki67^+^CD8^+^ cells (absolute number of Ki67 positive CD8^+^ cells per mm^2^) reflects both the absolute number of CD8^+^ cells and the percentage of Ki67^+^CD8^+^ cells. This parameter may be useful to predict the number of CD8^+^ cells that can be expected in a cancer in a given amount of time. Hypothetically, such information could be instrumental for an optimized timing of treatments with immune checkpoint inhibitors.

In summary, these data show that the fraction of proliferating CD8^+^ cells is usually below 3% in healthy secondary lymphoid organs. This rate varies markedly in inflammatory and neoplastic diseases. The striking link with favorable tumor features in colorectal cancer suggests a potential prognostic and clinical role of assessing Ki67^+^CD8^+^ cells.

## MATERIALS AND METHODS

### Tissues and tissue microarrays

Formalin-fixed paraffin-embedded tissue samples of 868 patients were selected from the archives of the Institute of Pathology of the University Medical Center Hamburg-Eppendorf, Germany. Conventional large sections were taken from histologically normal human tonsil (n=1), lymph node (n=1), thymus (n=1), ileum with Peyer’s patches (n=1), spleen (n=1), and appendix (n=1) as well as from lymph nodes containing breast cancer metastases (n=2). In addition, three preexisting TMAs were utilized for this study [35, 43]. An “inflammation microenvironment TMA” contained extra-large tissue samples (diameter: 4mm) from Hashimoto thyroiditis (n=2), Lichen sclerosus of the penis (n=2), sarcoidosis (n=2), sialadenitis (n=2), IgG4 pancreatitis (n=2), Crohn’s disease (n=2) and eczema (n=1). A “cancer microenvironment TMA” contained tissue samples also measuring 4mm in diameter from colorectal cancer (n=10), melanoma (n=2), prostate cancer (n=2), urothelial carcinoma of the bladder (n=2), renal cell carcinoma (n=2) and lung cancer (n=2). In addition, a colorectal cancer TMA was used containing samples measuring 0.6mm in diameter from 826 patients distributed across two TMA blocks. Available histopathological data for these cancers included pT, pN, tumor localization and immunohistochemically determined mismatch repair (MMR) status. The use of archived diagnostic left-over tissues for manufacturing of TMAs and their analysis for research purposes has been approved by local laws (HmbKHG, §12,1) and by the local ethics committee (Ethics commission Hamburg, WF-049/09). All work was performed in compliance with the Helsinki Declaration.

### Immunohistochemistry

Freshly cut 4μm tissue sections were used for fluorescence multiplex immunohistochemistry (IHC) analysis. Antibodies against Ki67 (Cat. #DIA-670-P1, Dianova, mouse monoclonal antibody) and CD8 (Cat. #DIA-TC8, Oncodianova, mouse monoclonal antibody) were used to identify proliferating cytotoxic lymphocytes (Table 5). The OPAL dye kit (Cat. #OP7DS1001KT, PerkinElmer, Waltham, Massachusetts, United States) was used for antibody detection. The experimental procedure was according to the manufacturer’s instructions (PerkinElmer). Slides were initially boiled in a microwave oven (15 minutes at 100° C in pH9 buffer) for antigen retrieval. Antibodies to Ki67 and CD8 were combined with DAPI staining in each experiment. One circle of antibody staining included peroxidase blocking, application of the first primary (Ki67) antibody, detection with a secondary HRP-conjugated antibody, fluorescence dye detection, and removal of the bound antibodies by microwave treatment (15 minutes at 100° C). This cycle was repeated for the second primary (CD8) antibody. Slides were subsequently counterstained with diamidinoino-2-phenylindole (DAPI) and mounted in antifade solution.

**Table 5:** List of the used antibodies, antigen retrieval (AR), dilutions, and opal dyes for multiplex fluorescence immunohistochemistry.

**Antibody target**	**Identifier**	**AR (pH value)**	**Dilution**	**Staining position**	**Opal dye**
Ki67	Dianova, Clone: Ki-67P, Cat#: DIA-670-P1	9.0	1:200	1	520
CD8	Oncodianova, Clone: TC8, Cat#: DIA-TC8	9.0	1:200	2	690

(AR = antigen retrieval, RTU = Ready to use).

### Quantification of proliferating CD8^+^ lymphocytes

Digital images of fluorescence stained slides were acquired with a Leica Aperio VERSA 8 automated epifluorescence microscope. Image analysis was performed using Image Scope software package (Leica Microsystems Wetzlar, Germany) and included segmentation of individual cells to detect expression of Ki67^+^ and CD8^+^ cells. A visually adjusted intensity threshold was set for each marker. Cells showing staining intensities above this threshold were considered “positive”. Representative analysis results are shown in Figures 1 and 2 as well as in Supplementary Figure 1. Biologically relevant tissue compartments were manually annotated by a pathologist (e.g. germinal centers, marginal zones and interfollicular areas in tonsils and lymph nodes, tumor centers and invasive margins in cancers, intraepithelial and subepithelial areas in inflammations). All areas matching compartment specific histology and sufficient tissue quality were included. For TMA analysis, spots were automatically identified and segmented with the HALO™ (Indica Labs, US) TMA module. Two parameters were measured for every individual tissue compartment: 1. The density of CD8^+^ stained cells per square millimeter was calculated by dividing the number of CD8^+^ cells by the measured area of each tissue spot. 2. The density of Ki67^+^CD8^+^ cells was calculated by dividing the number of Ki67^+^CD8^+^ cells by the measured area of each tissue spot. 3. The percentage of proliferating CD8^+^ cells was calculated by dividing the number of Ki67^+^CD8^+^ double positive cells by the total number of CD8^+^ cells.

### Statistical analysis

JMP Pro 12 software package (SAS Institute Inc., NC, USA) and R version 3.5.1 (The R foundation) [44, 45] were used to plot the data and to perform the Pearson correlation analysis, to study associations between cell densities and the percentage of proliferating cells.

### Ethics approval and consent to participate

Usage of archived tissues has been approved by local laws (HmgKhG §12) and the local ethics committee (Ethics commission Hamburg, WF-049/09). All work has been carried out in compliance with the Helsinki Declaration.

### Availability of data and materials

The datasets used and/or analysed during the current study are available from the corresponding author on reasonable request.

## Supplementary Material

Supplementary Figures
