# Supplementary material for: Prevalence of proliferating CD8+ cells in normal lymphatic tissues, inflammation and cancer
Source: Aging (Albany NY). 2021 Jun 3;13(11):14590–603. doi: 10.18632/aging.203113 (PMC8221353; doi:10.18632/aging.203113)
Supplement: Supplementary Figures [file aging-13-203113-s001.pdf]

SUPPLEMENTARY FIGURES

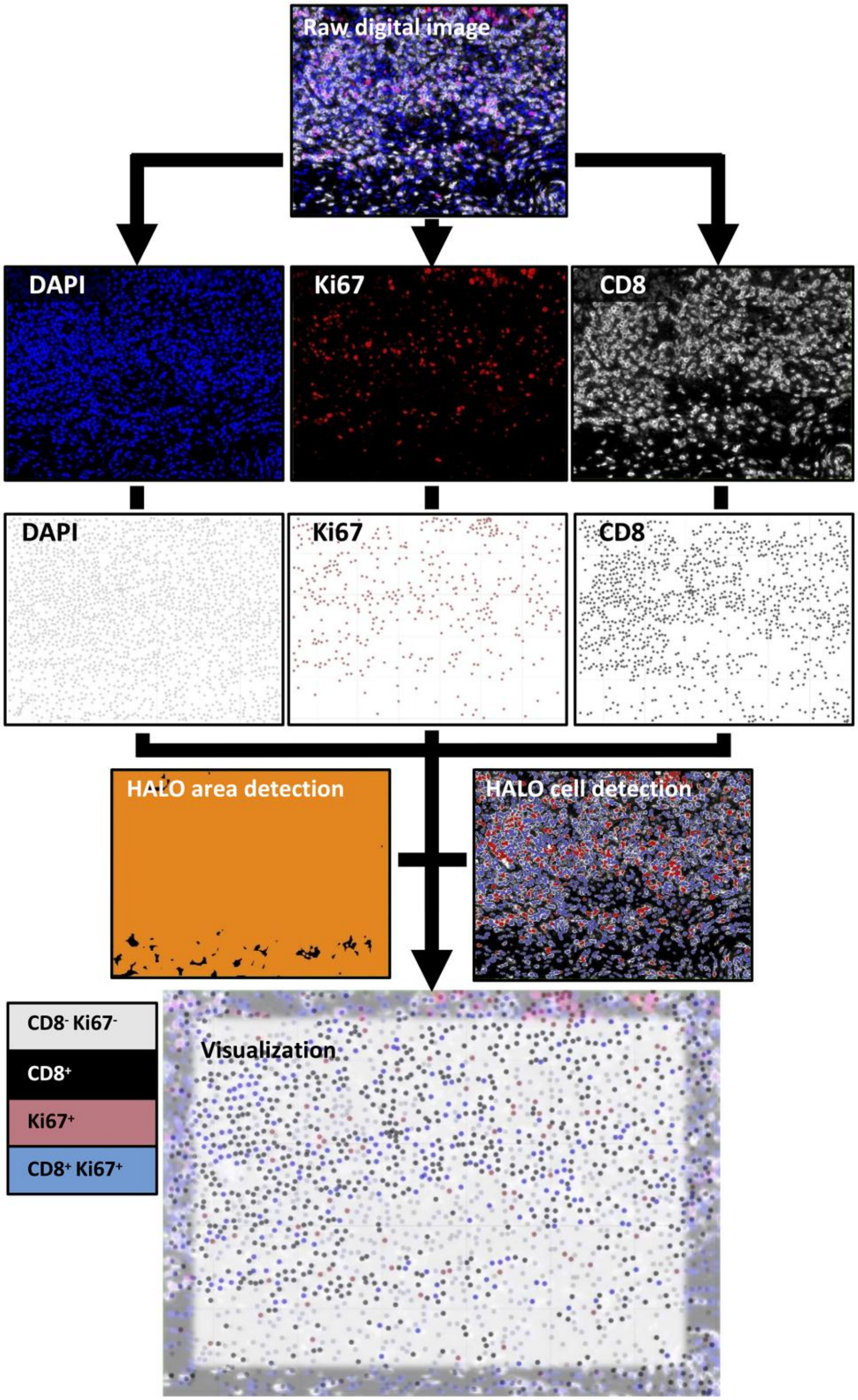

Supplementary Figure 1. Digital image analysis workflow. Area detection (orange), cell segmentation and the visualization of detected DAPI+ (grey), Ki67+ (red), CD8+ (black) and Ki67+CD8+ cells (blue) is shown.

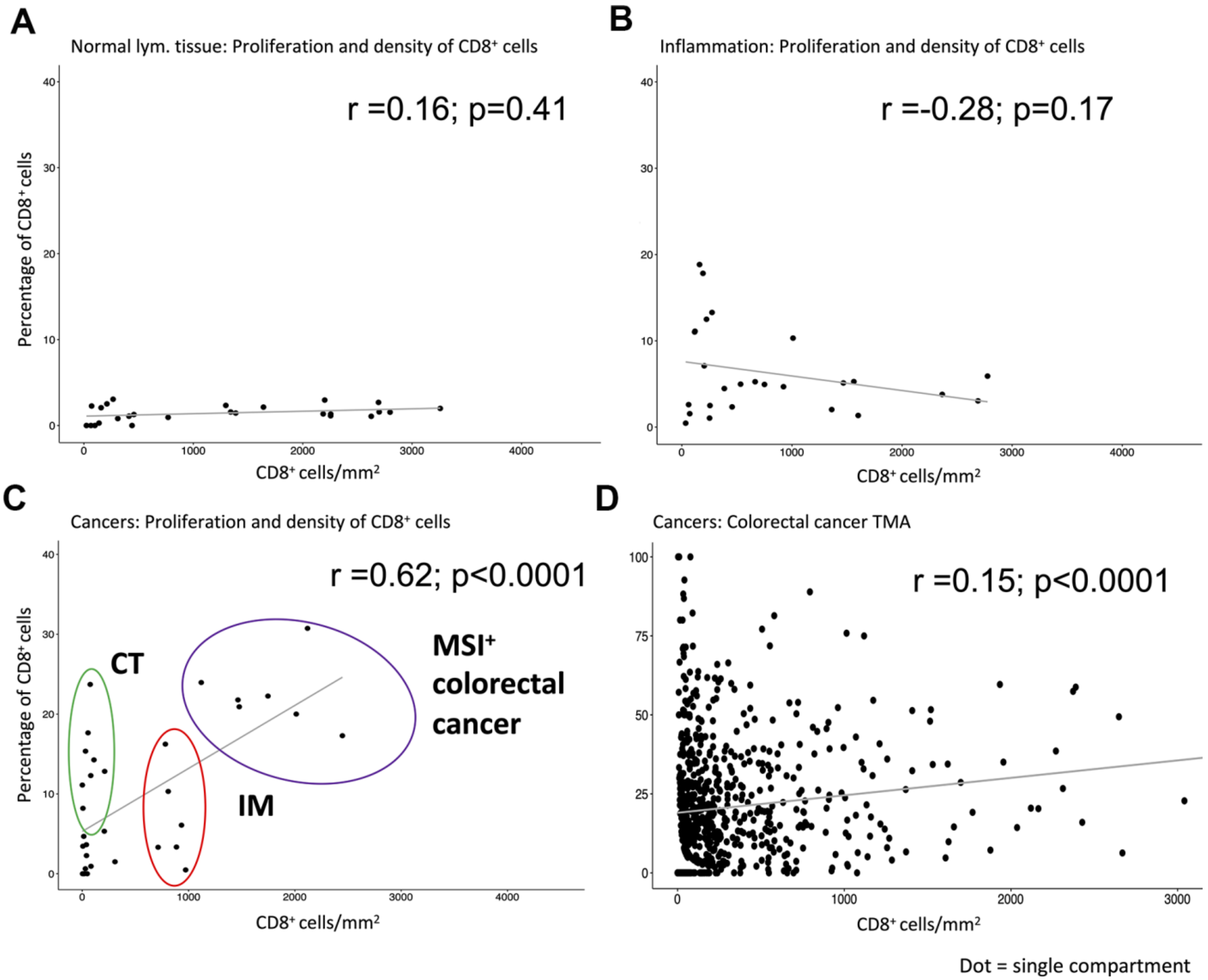

Supplementary Figure 2. Correlation of CD8⁺ cell density and proliferation rate in cancer. Pearson correlation analysis of the CD8⁺ density and the percentage of proliferating CD8⁺ cells in all analyzed compartments of (**A**) normal lymphatic tissue (n=26), (**B**) inflammations (n=26), (**C**) various cancer entities (n=41) and (**D**) colorectal cancer (n=765). r, correlation coefficent; p, p-value; CT, center of the Tumor; IM, invasive margin.
